# Opportunities and challenges with the German act for the protection of children with variations of sex development

**DOI:** 10.1038/s41443-022-00614-z

**Published:** 2022-10-05

**Authors:** Limor Meoded Danon, Katinka Schweizer, Barbara Thies

**Affiliations:** 1 Azrieli Faculty of Medicine, Bar-Ilan University, 8 Henrietta Szold St, Safed, Israel; 2 Department of Psychology, MSH Medical School Hamburg, Am Kaiserkai 1, 20457 Hamburg, Germany; 3 Institute of Educational Psychology, TU Braunschweig, Bienroder Weg 82, 38106 Braunschweig, Germany

**Keywords:** Quality of life, Risk factors

## Abstract

In May 2021, the German parliament passed a long-debated law to protect children with variations of sex development/sex characteristics from medically unnecessary surgeries until they are old enough to decide for themselves. This law joins similar laws passed in other countries in recent years and recognizes the rights of people with variations of sex development to self-determination and bodily autonomy. In this article, we discuss the notion of bodily autonomy and examine details of the German legislation in the context of psychosocial care. We focus on the following questions: (1) How may the law help to preserve the genital integrity and future bodily autonomy of newborns with variations of sex development (VSD)? (2) What are the opportunities and challenges of this law? (3) What strategies are needed to implement the law in ways that include medical professionals’ knowledge and skills, parental cooperation, and protection for the genital integrity as well as the future genital autonomy of newborns with VSD? We make two main arguments. On the one hand, this law has created a space for a new discourse and discussion on VSD in German society and enables the “wait and see” approach. This approach challenges the traditional “psychosocial emergency” policy aimed at quickly “repairing” atypical genitalia. On the other hand, the law is characterized by significant challenges. For example, it does not address the meaning of bodily autonomy in the context of newborns and their families with VSD, and it overlooks the important distinction between genital appearance, genital function, and gender identity. We offer various educational strategies that can be implemented with different target groups in Germany to meet these challenges and ensure the adequate implementation of this law.

## Introduction

The struggle for bodily autonomy and social recognition for intersex people and/or people born with variations of sex development (VSD) is a long and controversial one that touches on many sociocultural and psychological aspects of human life and experience. From the sociological and biopolitical perspectives, bodily autonomy for intersex people/people with VSD raises many issues regarding the different meanings of biological sex. The relationships between bodily sex characteristics and how one is, or should be, categorized in terms of either sex or gender attribution are not self-evident scientific facts that can be revealed only through the biomedical gaze. Rather, these relationships are socially constructed and dynamically change in different cultures, social interactions, performances, times, and spaces [1–7]. Nevertheless, (“Intersex” is still used as an umbrella term for various congenital conditions and sex characteristics, such as sex chromosomes, gonads, and reproductive organs, that are different from those of so-called typical male and female bodies. The term “intersex” is controversial among biomedical professionals as well as among people born with variations in sex characteristics and their parents, and is mainly based on the assumption that the word intersex implies a queer identity that undermines social norms and the differentiation between the two (presumed sole) sex/gender categories. Many scholars have referred to the controversy surrounding intersex and the politics of naming. See [8].) Bodies with VSD (in light of the terminological dispute over the pathologizing medical term “disorders of sex development” and the activists’ term “intersex,” we decided to use the term “VSD,” which focuses on the body and its sexual variations and includes people born with such conditions who are not aware of the term “intersex” or the controversies surrounding their bodies.) are usually perceived by medical professionals as pathological bodies that deviate from the supposedly natural social-bodily order, in which the biological sex of individuals develops along two distinct paths and gender identities signify (different) sexual characteristics [8–16]. Moreover, the geneticization of the medical field and the use of advanced biogenetic diagnostic technologies to identify the genetic etiology of variations of sex development narrow the meanings and essences of biological sex to the genetic sphere and preserve the pathological view of VSD.

Since the 1950s, the birth of a child with VSD has been perceived as a psychosocial emergency and treated according to the so-called “optimal gender policy” (OGP) paradigm. In this paradigm, early genital surgeries are thought to help babies with genitalia perceived to be atypical vis-à-vis dominant norms for categorizing bodies in terms of sex (i.e., two exhaustive and mutually exclusive categories: male or female) to be socialized as “normal” boys or girls and thus avoid anticipated social stigma and harm [17–20]. The premises of this approach remain embedded in medical care, and the idea of “successful sex assignment” is still implicitly and explicitly conveyed by medical guidelines around the world [21–24].

Many biomedical experts are convinced that their role is to heal the social bodies of patients with VSD, that is, to create bodies with less “uncertainty” around physical sex, where such uncertainty has been hypothesized (albeit without robust supporting evidence) to reliably lead to seriously adverse social consequences for the individual. The goal, then, is to enable young children and future adults to engage in social interactions without physical conditions that might cause shame, social stigma, or alienation. Yet, paradoxically, when people with varied intersex traits shared their experiences in the media, they reveal common traumatic experiences which they relate to negative consequences of early medical interventions. These consequences may include a lack of genital sensation, scarred and mutilated genitals; repeated “corrective” genital surgeries; and living in secrecy, shame, and social isolation [17, 18, 25–34].

The struggle for bodily autonomy for people with VSD—that is, the struggle to be allowed to make their own informed decisions about whether to undergo medically unnecessary genital surgeries when sufficiently autonomous—began in the 1990s. This struggle drew attention to the ways in which the OGP and “social emergency policy” violate their human rights, including their ownership of their bodies, their ability to protect their bodies from unwanted intrusions and changes and to actively consent to medicalized treatment, and their right to self-determination [35–38]. Since there have been no significant, or only minor, changes (e.g., alleged improvements in surgical techniques) in the treatment of infants born with VSD, especially those born with external genitalia judged to be markedly atypical, VSD is usually perceived as a socio-medical pathology that needs to be addressed as soon as possible through medical interventions. Such interventions continue to include genital surgery, removal of internal organs (uterus, gonads), and hormone therapy [39].

Intersex activists in particular have decided to turn to law enforcement and focus on human rights violations to create change. Pioneering laws have been passed recently in Malta (“AN ACT [*sic*] for the recognition and registration of the gender of a person and to regulate the effects of such a change, as well as the recognition and protection of the sex characteristics of a person”), (Gender Identity, Gender Expression and Sex Characteristics Act https://legislation.mt/eli/act/2015/11/eng/pdf) Portugal (“Right to self-determination of gender identity and gender expression and for the protection of each person’s sexual characteristics”), (https://dre.pt/pesquisa/-/search/115933863/details/maximized), and Germany (on which we focus here in detail) (https://dserver.bundestag.de/btd/19/246/1924686.pdf) to protect children with VSD from so-called normalizing surgeries and to recognize additional legal categories relating to gender such as “diverse” and “open” to provide more possibilities for gendered self-identification in relation to one’s sex characteristics [40] (The list of countries that legally recognize non-binary genders (i.e., which have additional options for gender categorization besides only male and female) can be found here: https://en.wikipedia.org/wiki/Legal_recognition_of_non-binary_gender#cite_note-19. We note that the conceptual and practical relationships between gender and sex are complicated, and not only in the context of persons with intersex traits. In fact, most such persons conceive of themselves as members of the dichotomous male or female sex category to which they were assigned at birth (often with surgical and hormonal reinforcement), and, moreover, identify with the social gender category that is normatively associated with their birth-assigned sex (i.e., cisgender). Other persons with intersex traits, by contrast, conceive of themselves as neither (exclusively) male nor female—or both male and female—in terms of sex, with varied self-identifications for gender that may include binary concepts such as man or woman, as well as non-binary concepts such as genderqueer, among others. Thus, there is no necessary or consistent relationship between a person’s physical sex characteristics, their sex assigned at birth, the sex by which they come to self-identify, or their associated gender identity). Nonetheless, despite growing recognition by various UN committees, new laws, and international legal bodies that aim to protect the child’s (future) right to bodily autonomy, the struggle to secure this right in practice continues with no significant change [41]. Even in Malta and Portugal, which passed laws to protect infants with VSD from harmful surgical intervention until they are developmentally capable of giving their own consent, surgical interventions continue, whether within the country itself or via medical tourism through which parents take their children to other countries for surgery (The report of the international NGO of intersex human rights which relates to the consequenses of Malta’s law: https://intersex.shadowreport.org/public/2019-CRC-Malta-NGO-Zwischengeschlecht-Intersex-IGM.pdf).

There are several reasons why the struggle for bodily autonomy has not yet achieved its goals and there remains a gap between the rights of patients who seek to end non-consensual surgical and hormonal interventions and the attitudes and practices of physicians. This situation may be due to a lack of doctor–patient–parent-related communication skills and a common language that addresses the physical characteristics of a newborn with VSD without judgmental or pathologizing terms. Timmermans and colleagues [42] raised questions regarding the knowledge and information transmitted between doctors and parents in the consulting room. Their study revealed how the dialog on genital surgery between parents and doctors revolves around the uncertainty that surrounds the children’s future, especially when surgery is not chosen, and that surgeries are the option most often chosen to reduce this uncertainty. In addition, the study showed that physicians are forced to cope with external pressure from human rights organizations in the United States and around the world that seek to prevent unnecessary genital surgeries but do not understand or want to understand the essential meaning of this pressure. That is, most physicians are detached from the historical, social, and physical contexts of intersex adults who have been harmed by surgical interventions.

Another reason for the ongoing struggle for bodily autonomy is related to the binary body-gender model, which is firmly embedded in societal and medical thinking [43, 44]. Uncertainty regarding bodies with VSD, especially those with genitalia judged to be markedly atypical, stems from the assumption that the normative socialization process in which children identify with feminine or masculine gender traits is possible only if genitalia are judged to fall within dominant standards for binary male/female categorization. Therefore, it is believed that if genitalia judged to fall outside these standards, it will distance children from the (hetero- normative) social order.

Third, the line between “necessary” and “unnecessary” medical or surgical intervention in relation to genital cutting is ambiguous, open to interpretation, and lacking consistency [45–48]. The common agreement on necessary surgeries in the context of VSD, is that it is saving lives, preventing serious health risks that cannot be as safely or effectively addressed non-surgically, such as, removing gonads with cancer or a high risk of developing cancer in the near-future, modifying the urethral opening (not necessarily at the tip of the penis) to enable urination, and preventing infections of internal organs. However, in the context of genital surgery in infants, such as vaginoplasty, vaginal enlargement, clitoroplasty, separation of the urethral and vaginal openings, and hypospadias surgeries to move the urethral opening to the tip of the penis, the necessity of these surgeries is highly debatable. What are the purposes of these surgeries? How is their success measured?

The answers to these questions are not simple and sometimes depend on one’s position and interests within the dynamic between doctor-baby/patients-caregivers/parents. For instance, parents and doctors might be motivated to alter genital appearance according to their own (socio-cultural) perceptions and experiences with genital appearance and gendered behaviors. For example, they might believe that in order to be “proper” boys their sons should be able to urinate standing up; or they might believe that “proper” girls should have “petite” clitorises, small labia and open vaginas that allow penile penetration. Although these are not strictly physical health issues, parents may nevertheless feel that such psycho-social motivations are sufficient justifications for surgery. However, babies and toddlers are too young to consider or compare their bodies/genitals to social norms/order. Moreover, lessons must be taught from adults born with VSD who experience and perceive their surgeries as having failed and/or damaged their bodies irreversibly, in turn affecting their living experiences negatively, and leading to additional surgeries that only increased their genital differences.

Here are some examples of the gap between these two viewpoints from a study conducted by one of the authors (LMD). The study highlights the discursive gaps between medical experts and people with VSD in Israel [49]. In the following excerpt, one of the most experienced endocrinologists in Israel, describes the reason for early genital surgeries in his view and explains how he relates to parents who consider delaying early surgical intervention:

*You can determine gender at a young age, before socialization at preschool, and once you have determined it at a young age, you prevent many of the problems that can develop … You want to have a boy who can urinate and feel normal around others, so we talk to the parents, we ask [questions]. If the parents say “Listen, we don’t care,” no doctor will influence them in any way, but he will tell them, “Listen, you are very smart parents, but it is very bad for your child. He’ll be very uncomfortable in first grade or third …” The pediatrician knows much better than the inexperienced parent who comes at age 28 and says, “I’m being influenced by someone.” The doctor has a great deal of experience in society. He has been a pediatrician for many years and knows better than the parents of a first child*. (Prof. Ziv, 24.11.19) (All the participants’ names have been changed to protect their privacy).

Assaf (40 years old) was born with minor hypospadias and underwent surgery to construct the urethral opening at the tip of his penis at age two. When he was 16, the implanted urethra began to close, reducing the flow of urine and causing him physical and social suffering. He experienced urinary hesitancy, was ashamed to urinate in the company of others, and suffered from frequent urinary tract infections. From age 16 to 18, he underwent several surgeries. He learned that he would never urinate “normally” because his urethra could not be completely repaired. He was given a kit with a sterile tube to help him urinate if the constriction worsened. His daily routine as an adult revolved around urination issues. He needed 20 to 30 min to urinate, so he planned his meetings and social interactions according to his ability to hold his urine. After a traumatic experience at age 38 in which his urethral opening was completely blocked, he decided to put an end to his suffering and asked for his “natural” genitalia, the genitalia with hypospadias he was born with, to be reconstructed. Following is his description of the dialog between him and the doctor who operated on him in his youth:

*I told him all I wanted was for him to get me back to how I was born….I wanted to pee where the opening I was born with was, a few centimeters back. He told me I might have to urinate while sitting. I told him I’d been doing this for twenty years. He told me I would urinate in a nonuniform way. I told him okay, “I’ve been doing this for twenty years. To tell the truth, I don’t care how I pee. The main thing is to pee.”*(Assaf, 23.3.20).

These two excerpts illustrate both the gap between the different worlds of knowledge and expectations regarding the success of surgeries. In addition, these different perspectives and experiences reveal the physical and emotional layers, which do not always overlap, of the outcomes of genital surgeries in patients’ lives. These layers include genital functionality—the ways in which the genitals function as organs that can excrete waste—and have sensual and sexual implications (e.g., erection). Genital appearance does not necessarily reflect genital functionality. Moreover, the gaze on the genitals is neither fixed nor universal, but rather dynamically changes through social interactions and experiences, as studies have shown [50]. Another layer to consider is the connection between genitalia and gender attribution, i.e., the presumed uniform, immanent connection between genitalia and gender identity or expression, which reflects the automatic assumption that arises from the binary conception of both gender and sex that encourages rapid surgical intervention. But how exactly are genitalia and gender related? How can a baby’s gender be assessed? How can the process of his gender socialization be predicted?

Finally, the difficulty people with VSD experience in achieving bodily/genital autonomy in the medical sphere touches on deeper conflicts: such autonomy threatens medical power and control over the scientific knowledge of human bodies, biological sex, and dimorphic sex/gender relations. Historically, the medical system has established categories of biological sex and gender, and economic and cultural interests have played integral roles in this process [10, 11, 13]. Activists’ participation in human rights movements, alongside NGOs and laws that change medical policy, both aim to decrease the power of the medical system, especially its self-authorized “ownership” of defining sex and gender. Laws are intended to create change from the top down, through regulations that protect children’s (future) right to bodily autonomy. Yet, as mentioned, in Malta and Portugal the laws did not engender significant change in medical policy.

The German law, on which we focus here, is an example of a state regulation intended to suspend and regulate (medically unnecessary—that is, cosmetic or elective) genital surgery on newborns with genitalia judged to be atypical. We analyze the details of the new German law and focus on three questions. First, how does the law protect the future genital autonomy of newborns with variations of sex development (VSD)? Second, what are the blind spots in this law? Third, what strategies are needed to implement the law in a way that includes both medical professionals’ knowledge and skills and efforts to protect the future genital autonomy of newborns with VSD in a balanced manner? Before we address these questions, we review some milestones of the legislative process.

## The legislative process in Germany

The international intersex rights movement and the ongoing paradigm shifts that have been taking place thanks to former patients and experts with lived experience, particularly via the breaking of silence among people with VSD, have been central forces for social change in Germany. In general, patient-centered medicine has gained more relevance, and patient-led movements and improvements in patients’ rights have influenced legislation. An early development was the 2012 publication of a statement of the German Ethics Council, which acknowledged both (a) the need for the legal recognition of people born with diverse sex characteristics and (b) the ongoing human rights violations involved in the medical treatment of people with VSD [47]. Following this, two documents relevant to the field of medicine were published in two years. The German Medical Association published a statement on the treatment of patients with VSD in 2015, and, in 2016 the German Association of the Scientific Medical Societies (AWMF) issued new medical guidelines that were participatory and included patient groups and psychosocial professionals [48] (The participatorily developed AWMF S2k guidelines titled “Variants of Sex Development” from 2016 and the reports and studies of the Interministerial Working Group stress the need for readily available counseling for parents of children with VSD. They are currently in the process of revision). These guidelines were initiated by the German Urological Society and consented by representatives from the Surgical, Endocrinological and Psychosomatic Associations, and others such as the Society for Sex Research in collaboration with patient groups (CAH) and intersex advocacy groups (Intergeschlechtliche Menschen e. V.) They address the issues of early surgeries and warn doctors to carefully consider strict necessity on medical grounds.

In the aftermath of the Ethics Council’s statement, the medical discourse has begun to change and legislative processes have been initiated to improve the human rights and visibility of people with VSD and better represent them in public. Even before 2021, when the law for the protection of children with VSD was passed, far-reaching legal changes had taken place in 2013 and 2018, when two amendments to the Civil Status Act were implemented, allowing parents of newborns with VSD to choose “diverse” or “left open” as the legal gender category for their child, based on the idea that uncertainty regarding sex categorization entails a greater-than-usual uncertainty about subsequent gender identification (which is otherwise presumed to correspond to sex in the conventional binary manner). The amendments also enabled German citizens who can testify or give proof of medically confirmed VSD to use these additional categories for themselves.

Thus, German gender recognition and civil status now includes four gender categories: female, male, diverse, and open. Legal recognition of gender diversity is important for many people, including transgender persons (who may or may not have a VSD, but most commonly do not), but it is not as obvious that this recognition is helpful to children with VSD (whose subsequent gender self-identification cannot be known). Will it protect these children from unnecessary medical intervention? The Law for the Protection of Children with VSD (Unlike English and some other languages, German has only one word, *Geschlecht*, to cover both psychosocial gender and somatic sex characteristics.) was debated in Germany beginning in October 2018, when a one-day interdisciplinary conference was held in Berlin, hosted by the Federal Ministry of Justice and Consumer Protection. This meeting included various experts as well as people with VSD and parents of children with VSD. The main controversies revolved around the need for medical treatment and the underlying categorization of male, female, and “different” bodies. In relation to “different” bodies, genital surgeries such as urethral relocation for hypospadias are medically “justified” for babies with 46,XY karyotype, and, vaginoplasty and clitoroplasty are “justified” in infants with 46,XX karyotype with congenital adrenal hyperplasia (CAH). These two conditions, hypospadias and CAH, are regarded as among the most common and well-known VSD conditions. From the beginning of the debate, these two groups of conditions were at the center of the heated controversy over genital autonomy. After a period of public silence, the so-called “big coalition” of the two political parties that formed the federal government (the Christian Democratic Union and the Social Democratic Party) from 2017 to 2021 kept their political promise to initiate legislation to better protect bodily integrity and the (future) bodily autonomy of all children born with VSD. They circulated a draft of the law in the winter of 2019 among relevant stakeholders, and advocates were invited to comment. The German Society for Sex Research (Deutsche Gesellschaft für Sexualforschung, DGfS), for example, pointed to the importance of protecting the right to bodily integrity, the future bodily autonomy and self-determination of all children. Other statements, for example that of the German Medical Association, were critical and did not see the need for specific legislation or legal supervision, especially after various medical societies had already agreed upon the S2k guidelines (described above). The doctors argued that they did not need legal supervision. Other groups argued for a law that would apply to all children and adolescents (defined as under-18-year-old), including gender-variant children and teens.

After the first draft was revised, a hearing took place in January 2021 with various experts (Among the multidisciplinary experts were two psychologists, including K.S., one of the authors of this article, two legal experts, two psychologists, and three medical experts, one of whom represented the German Medical Association (Bundesärztekammer)). The law was passed shortly thereafter, in May 2021. No official English translation is available at this time (Here is the link to the final version of the law: https://www.bgbl.de/xaver/bgbl/start.xav?startbk=Bundesanzeiger_BGBl&start=//*%5b@attr_id=%27bgbl121s1444.pdf%27%5d#__bgbl__%2F%2F*%5B%40attr_id%3D%27bgbl121s1082.pdf%27%5D__1634769416615). In the following section, we cite passages of the law and address the main issues that arise from them (to avoid authors’ subjective interpretations in the translation process, we used the deepL translation tool).

## Questions, strengths, and limitations of the law

Does the German (DeepL is an open source translator (https://www.deepl.com/de/translator).) law for the protection of children with variations of sex development bridge the gaps between people with VSD/intersex/and the medical system? How, if at all, does it address issues related to communication and biomedical framing? Does it differentiate between necessary and unnecessary medical and surgical intervention? How does it balance or preserve the power of the medical system? Which chances for better health care does the law transport into the medical field? In this section, we address some of these questions in an attempt to examine this law’s main strengths and weaknesses.

Like the Maltese and Portuguese laws, the new German law has significant strengths. First, it recognizes the social and legal existence of children with VSD. Second, it seeks to protect children with VSD from irreversible physical and emotional harm. Third, it acknowledges the rights of children with VSD to have future autonomy, especially to consent, when they are old enough to make informed decisions regarding their bodies. Fourth, as it is very specific in its requests for an optional interdisciplinary commission, if implemented consequently, it could even contribute to an improvement of true multidisciplinary VSC/intersex/health care specifically regarding information and communication with parents. Finally, it acknowledges the differences between sex characteristics and gender.

Nonetheless, an examination of the main sections of the law under the title “the treatment of children with VSD” (Article 1) reveals several open questions and challenges that could also weaken the law’s power to address its aims.

The law consists of five articles. The first article contains the law’s substantive provision (actually amending the Civil Code [introducing § 1631e Bürgerliches Gesetzbuch]). Articles two to five contain transitory and procedural provisions. The first paragraph opens with the following statement regarding parental custody:

*Parental custody does not include the right to consent to the treatment of a child with VSD who is incapable of giving consent or carrying out such treatment themselves, which, without any further reason for the treatment being added, is carried out solely with the intention of bringing the child’s physical appearance into line with that of the male or female sex and gender*.

In the extended version of the law, VSD is introduced as an umbrella term for an “incongruency” of the sex classification, including the chromosomal, gonadal, hormonal, or genital characteristics. This description is based on the German Urological, Surgical and Endocrinological Association’s guidelines. Furthermore, in this section, the text does not distinguish between different categories and diagnostic groups included under the umbrella of VSD but implies the existence of a gap between the rights of children with VSD and the interests of those who care for them and raise them (parents) with regard to body-gender issues and dynamics. Unlike the Maltese and the Portuguese laws, the German law highlights the importance of the parents’ role as decision makers for their children, in allowing their children to receive treatment aimed at physical–gender alignment or preventing such treatment, for example.

Furthermore, in Paragraph 2, the law addresses the issue of surgical interventions and again emphasizes the parental role and involvement in making decisions regarding surgical procedures and consent:

*In surgical interventions on the internal or external sexual characteristics of a child with VSD who is incapable of consenting, which could result in an approximation of the physical appearance of the child to that of the male or female sex and for which the authority to consent is not already lacking under Paragraph 1, the parents may only consent if the intervention cannot be postponed until the child has made a self-determined decision*.

Paragraph 2 can be seen as the core of the new law. It implicitly states that any sex-assigning or aligning interventions are to be postponed until the child can consent itself in person. It focuses on regulating and preventing traditional surgical procedures aimed at “normalizing” the bodies of children with VSD and denies parents’ right to consent (that is, to give “proxy” consent on the child’s behalf) to such surgeries. At the same time, this section does not specify which VSD conditions or physical conditions related to VSD require urgent, necessary surgeries and which do not. Parents can only consent to urgent, necessary surgeries that cannot be delayed until the child consents.

In Paragraph 3, the law states that when parents seek to consent to a surgery aimed at creating physical-sex/gender alignment, they must obtain the approval of the Family Court. Parents need to obtain a decision from an Interdisciplinary Committee that “presumes that the planned intervention is in the best interest of the child.” This section of the law seems to aim at slowing down the rapid decision-making process regarding the performance of surgeries aimed at normalizing children. It shifts the responsibility from parents to an Interdisciplinary Committee that has to be set up for discussing why the requested surgery is necessary now and why it cannot be postponed. The law explicitly states who is eligible to become a member of the interdisciplinary committee and which contents the decision statement needs to include. However, several issues are left unclear. For example, how will the Interdisciplinary Committee monitor/regulate itself? How does it differ from the clinical “DSD multidisciplinary teams” that already exist in some centers of expertise in Germany? The law specifically describes, in Paragraph 4, which professionals should serve on the Interdisciplinary Committee (Table 1), [23]. The members of an interdisciplinary commission board are listed in Table 1.

**Table 1:** Members of the interdisciplinary commission board.

1. The responsible physician, who treats the child
2. Another medical person
3. A person with a psychological, child/youth psychological or psychiatric professional qualifi cation
4. A person trained in ethics
5. A peer-to-peer- counselor (if parents wish for them)

A central question is, how can the law ensure that this committee adopts diverse discourses and not, as studies have shown is often the case [51–53], be dominated by the biomedical discourse? To avoid such a situation, the law suggests that the parents may request to add to the Interdisciplinary Committee “a counselor with a variation of sex development.” Appointing peer-to-peer counselors with VSD to the Interdisciplinary Committee is an important and innovative move that may allow its members to learn through these counselors’ personal experiences about optional outcomes of a sex-assigning medical intervention. However, it is unclear why the law assumes that parents will invite counselors with VSD if there is a possibility that these counselors may challenge the parental quest for approval for “corrective” surgeries. Nevertheless, in Paragraph 5, the law addresses the contribution of a peer-to-peer counselor, such as a person with the same VSD condition of the case discussed, to be included in the content of the Interdisciplinary Committee’s reports to the Family Court when requesting to perform surgeries on babies/children with VSD.

The relevant contents of the statement of the interdisciplinary commission are presented in Table 2.

**Table 2:** Content and Checklist: Mandatory Aspects that the Interdisciplinary Committee must present and sign for the Family Court when requesting to perform surgeries on babies/children with VSD.

1. the name and designation of the members of the Interdisciplinary Committee and their qualifications,
2. the child’s age and physical condition;
3. the name of the planned intervention and the indication/s for it;
4. the reasons for the intervention, taking into account the best interests of the child, and, in particular, the risks associated with this intervention, with other treatments, or with not having an intervention until the child has made a self - determined decision;
5. whether and by which Interdisciplinary Committee members a discussion was held with the parents and the child and whether and by which members the parents and the child were informed and advised on how to address the specific VSD;
6. whether counseling of the parents and the child by a counselor with VSD has taken place;
7. the extent to which the child is capable of forming and expressing an opinion *and whether the planned intervention corresponds with the child’s will*; and
8. whether a counselor with VSD was involved *and concurs with the statement*.

The content that should be presented in a statement to the family court (in Table 2) by the Interdisciplinary Committee is significant. If implemented accordingly, the content to be documented could serve as a checklist and can contribute to increasing the transparency of the decision-making process not only for the court but also for the children, who, in the future, will have access to the documentation of the process of decision-making and consenting to irreversible interventions. The relevant medical records, documentation and the content of the interdisciplinary committee’s statement are to be kept and preserved until the child reaches the age of 48 years. This is also a new regulation by the new law.

The members of the Interdisciplinary Committee are also aware that their signatures and involvement are intended to minimize risks, prevent irreversible harm and to take all possible risks into account. The Family Court has the exclusive authority to approve or not approve to a medical intervention after reviewing the Interdisciplinary Committee’s statement.

It will be interesting to examine in the future which surgeries are approved by the Interdisciplinary Committee and which, if any, are rejected by the Family Court. Will the Family Court serve as a rubber stamp for the Interdisciplinary Committee’s decision or as an independent critical organ? Moreover, it seems that the law deliberately refrains from clearly stating which surgeries are performed for cosmetic, “corrective” reasons, that only the children themselves, when they will be old enough and able to consent in the future, will decide if they want to go through or not. Further, the law refrains from explicitly describing which surgeries are subject to the Interdisciplinary Committee’s review and which are not. Also, the specific kinds of damage that the medical procedures are supposed to prevent are not explicitly addressed. For example, how can the Family Court assess the emotional and physical damage that may result from a clitoroplasty, a vaginal opening procedure, or a hypospadias “repair”? In addition, the law ignores the ways in which repeated invasive medical examinations may also cause physical and/or emotional damage to babies and children with VSD.

These open questions, which we also carefully like to address as “blind spots” in the law, touch on the same issues and tensions that remain open among medical experts and activists. It will be an important task for the evaluation of the law as well as the field of intersex studies to follow and examine how the care takers involved, medical professionals, parents and the court implement and interpret the law, based on real life experiences and interactions.

## How to implement the German law?

Aside from the case management that the law refers to, there are social issues that must also be addressed. As noted above, among the reasons for the gap between the struggle for preserving bodily integrity of people with VSD and the continuing, often unnecessary medical interventions performed non-consensually, are missing or problematic communication styles and the lack of distinction between necessary and unnecessary surgeries. For parents to be more involved and better informed about the implications of such procedures, they must have access to information regarding the outcomes of both surgeries and hormone treatments and the risks and benefits of their postponement.

As studies show [45, 54–56], there is a lack of evidence-based data on the short- and long-term physical and emotional implications of necessary and unnecessary surgeries performed on children with VSD. In light of this uncertainty, how can parents make the right decisions for their children? As Streuli et al. [57] argue, “[t]he lack of detailed objective criteria, such as a quantifiable probability that a particular patient will lead a happy life after genital surgery, generates systemic weighting biases, giving certain cues (such as standing to urinate or having heterosexual intercourse) too much or too little weight.”

The existing uncertainty might contribute to maintaining biomedical professionals’ power to continue the same “corrective” medical practices. Thus, to achieve the law’s aim to protect children with VSD, the Interdisciplinary Committee must demand and present comprehensive information about each intended procedure, including short- and long-term data on the physical, emotional, and social risks and implications—from as many people as possible who have the relevant VSD condition—because each experience, each embodiment narrative might be different. The range of risks to name in the statement may vary. A comprehensive statement should be holistic and evaluate risks both of waiting and postponing vs. immediate intervening. Besides mere medical information it should also consider long-term consequences and outcome such as body-self relation and perception, shame and self-esteem in relation to genital appearance/function and the idea of developing a sense of “false self”, intimate interactions, parent–child–doctor relation and trust, etc. Only then will its members be able to assess whether or not the intended procedures are actually medically necessary at this point of time for the patients’ physical and emotional well-being.

Beyond the Interdisciplinary Committee’s work, and to help reduce parents’ uncertainty and fear concerning their children’s’ bodies with VSD as well as to highlight the significance of this law to the German public (and to the European and global public), it is necessary to act in several arenas simultaneously. First, sex and gender diversity need to be included in medical training, i.e., the two categories of male and female bodies must be broadened in the training of healthcare professionals involved in working with people with VSD. This can probably only be achieved when biomedical professionals, healthcare providers, and people with VSD work together to educate others. Moreover, educational modules and workshops for healthcare professionals, parents, and people with VSD are crucial. These modules will focus on the law’s contents and issues surrounding bodily /genital integrity of newborns with VSD. They will need to address the awareness of a positive language and a non-judgmental nor non-pathological VSD terminology; distinguish between necessary or essential medical procedures and unessential procedures that should be postponed or avoided; and challenge simplistic or unreflected assumptions about a default relationship between genital appearance and gender identities.

Second, to raise public awareness of the significance of the law, social media campaigns including e.g., podcasts and blogs on bodily integrity and the existence of VSD/intersex in general are needed, as well as on the medical history of sex, gender, and sexuality, and the living experience of people with VSD are of great importance. Third, the law also addresses the need to improve parental support and care for parents. Getting in touch with experienced parents and other people with VSD, e.g via a peer counselor could provide vital support and first hand information and help in overcoming taboos, silencing and isolation.

## Discussion

In the past 60–70 years, medicine (supported by the psychological field) has developed a policy of identifying an “optimal” gender for people with VSD/intersex by following the criteria of technical possibilities and social gender norms rather than the criteria of self-determination, an open future and life-long healthcare. Thus, a sex characteristic that appeared to be more typically female or male has been of higher value in the decision-making process than the patient’s personal right to decide. Today, the human rights perspective is gradually replacing optimal gender thinking. Nonetheless, due to the persistence of binary sex-gender assumptions not only in medicine but in society in general, this paradigm change is taking time and requires additional interventions that support caretakers in working with parents and families who have to decide for their children.

While some German physicians appear not to approve of the top-down paradigm of enforcing medical change, it may be useful for them to view this law as an anchor for the Hippocratic Oath that allows them to wait and think about their treatment decisions and actions, reflect on their existing knowledge, and examine or reexamine their attitudes and assumptions. The law creates a bureaucratic mechanism that requires doctors and various experts to examine the reasons for and against surgical intervention. The law allegedly embraces uncertainty and invites parents to raise, for instance, children with genitalia or gonads perceived to be atypical and to protect these children from physical and emotional harm. This is a challenging and courageous move for German society. Parents and doctors need to understand that feminine or masculine aspects of personality and the relationship between the genitalia are not predictable and not the main issue for children with VSD. Rather, transparency of information-giving regarding the consequences of surgical interventions, especially regarding risks and damages that may result from “corrective” surgeries, should be the main interest of parents and doctors. Furthermore, parents and doctors need to look primarily at the immediate needs of babies/patients, on the basic human ‘here and now’ needs, to be loved and cared for, to know and to feel they are significant, valuable and special to their primary caregivers, parents and families. Parents need to focus both on the presence and the future of their child, on daily parent–child interactions and needs and put aside their worries about their children’s sexual health, gender expressions, and identities, which should be open to the children to explore and experience themselves throughout life and in their social interactions throughout development.

One of the basic needs of children is to experience their bodies and their full potential and sensual aspects and not suppress them. Moreover, caregivers need to understand what motivates parents who request “corrective” surgeries. What are their fears? For parents, understanding the meaning of bodily integrity and autonomy means engaging with other parents and support groups available virtually and in person.

There are significant cultural gaps in the perceptions of bodies, gender, and sexuality. Therefore, the different meanings of these categories need to be discussed and assessed by parents themselves. The new German law is another historic milestone within a climate of a beginning significant change in the medical treatment paradigm. Its claim to document the process of decision-making and any details and reasons for not postponing sex-assigning surgery and interventions in children and its successful implementation could contribute to high quality health care standards. If medical caretakers will acknowledge and implement the laws’ tasks accordingly, some aspects, like the content-checklist for the interdisciplinary commission’s statement might become a helpful tool in care- and information- giving. Further, it might thereby also contribute to the process of acknowledging and reflecting bodily diversity and variations of sex development within the medical system, but also on a broader societal level. But to adequately introduce it to the German public, it is important to act in broader educational and social spheres. Integrating VSD/ intersex more visibly within sex education curricula in schools would be a first step.

## Data Availability

All data generated or analyzed during this study are included in this published article (and its supplementary information files).
